# Multitiered Proteome Analysis Displays the Hyperpermeability of the Rheumatoid Synovial Compartment for Plasma Proteins

**DOI:** 10.1016/j.mcpro.2024.100900

**Published:** 2024-12-31

**Authors:** Eva Maria Stork, Sofia Kalaidopoulou Nteak, Danique M.H. van Rijswijck, J. Mirjam A. Damen, Hans Ulrich Scherer, Rene E.M. Toes, Albert Bondt, Tom W.J. Huizinga, Albert J.R. Heck

**Affiliations:** 1 Department of Rheumatology, Leiden University Medical Center, Leiden, The Netherlands; 2 Biomolecular Mass Spectrometry and Proteomics, Bijvoet Center for Biomolecular Research and Utrecht Institute for Pharmaceutical Sciences, University of Utrecht, Utrecht The Netherlands; 3 Netherlands Proteomics Center, Utrecht, The Netherlands

**Keywords:** rheumatoid arthritis, plasma proteomics, synovial fluid, autoantibody repertoires, Fab profiling

## Abstract

Rheumatoid arthritis (RA) is characterized by synovial hyperplasia and cartilage/bone destruction. RA affects the synovial joints, the synovial lining, and the permeability of the synovium. As the latter is of central relevance for the distribution of systemically delivered therapeutics into synovial fluid (SF), we here assessed the protein composition of paired plasma and SF of patients diagnosed with RA at three distinct levels of depth using mass spectrometric approaches: the “total” proteome, the “total” immunoglobulin G1 (IgG1) antibody repertoire, and the RA-specific anticitrullinated protein IgG1 autoantibody repertoire. The SF proteome was found to be dominated in numbers and concentration by plasma proteins, although we additionally detected several cartilage- and neutrophil-derived proteins of lower abundance. Strikingly, the plasma proteins were not only qualitatively reflected in SF but also quantitatively, independent of their size and/or other biochemical features. Also, the synovial “total” IgG1 and autoreactive anticitrullinated protein antibody IgG1 repertoire highly resembled the IgG1 repertoires detected in plasma within the same patient. Our comprehensive multilayer data thus reveals that the proteome, including the dominant, most abundant (auto)antibody clones, present in SF of RA patients is a direct reflection of the proteome present in blood, spiked by the local (immune) processes within the RA joint. We thus conclude that proteins directly pass from blood into SF of these joints without substantial bias. These findings thereby not only exemplify the use of in-depth multilayer proteome analyses to revisit basic concepts underlying RA pathology and to monitor the local (immune) processes destructive to cartilage but also provide evidence indicating that (protein-based) therapeutics may equally enter SF of swollen joints and that pharmacokinetic analyses of such therapeutics in blood are directly relevant to the synovial compartment.

## Highlights

• Plasma and synovial fluid (SF) of rheumatoid arthritis (RA) patients were compared.
• Plasma and SF proteomes and (auto)antibody repertoires were analyzed using LC-MS.
• Synovial proteomes, IgG1 and ACPA IgG1 repertoires resembled their plasma equivalents.
• The RA synovial compartment hence showed to be hyperpermeable for plasma proteins.

## Graphical Abstract

**Figure:**
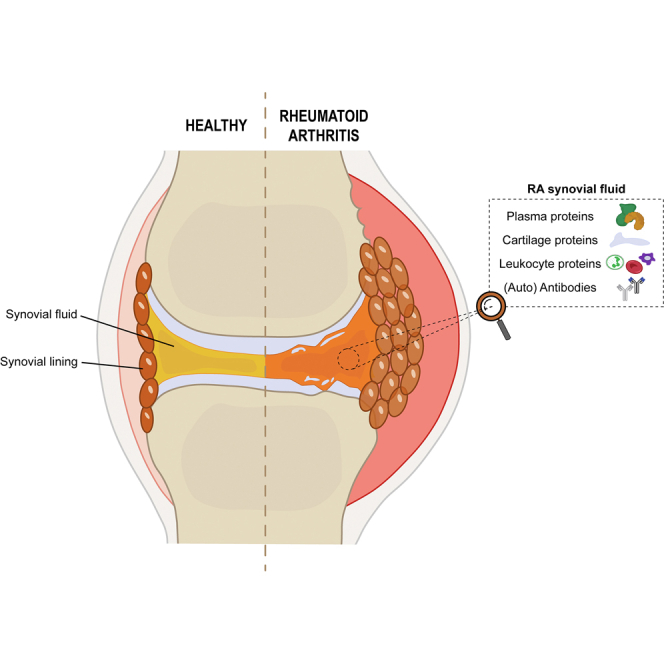

## In Brief

Here, we analyzed the protein composition of plasma and synovial fluid (SF) of individuals diagnosed with rheumatoid arthritis (RA). We measured proteins by mass spectrometry at three distinct levels, namely the overall proteome, the global immunoglobulin G1 clonal repertoire, and an RA-specific immunoglobulin G1 autoantibody clonal repertoire. Strikingly, the relative concentrations of each protein measured displayed a high correlation between plasma and SF, from which we conclude that in RA patients all proteins (including antibodies and autoantibodies) pass in an unbiased way directly from blood into the SF of their joints.

**Fig 1:**
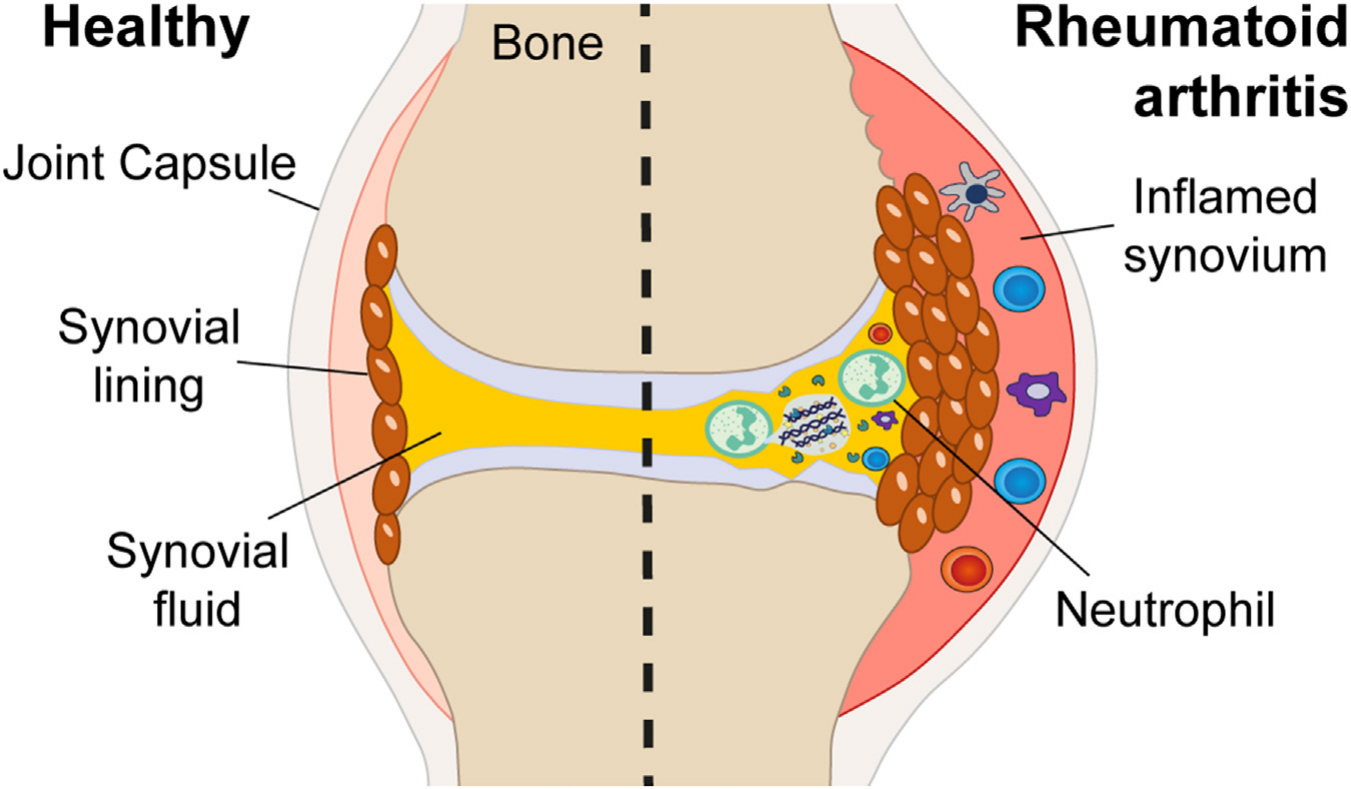
**Changes in the physiology of the joint from healthy (*left*) upon joint inflammation in rheumatoid arthritis (RA) (*right*), adapted from Smolen *et al.*** (1) **and Nygaard and Firestein** (4).

Rheumatoid arthritis (RA) is an autoimmune disease affecting about 0.5% of the population worldwide (1, 2, 3). Albeit RA is a systemic disease, it is primarily characterized by chronic inflammation of the synovial joints, which causes joint swelling accompanied by morning stiffness and tenderness on examination (4). Synovial joints, such as the knees, hips, hands, and feet, are surrounded by an articular capsule and the highly vascularized synovial tissue, which seals the joint cavity by a thin cellular layer called synovial lining (Fig. 1). The cavity within a synovial joint is filled with synovial fluid (SF), serving as joint lubricant and nutrient source for the nonvascularized articular cartilage (5, 6).

Under physiological conditions, SF sparsely contains cells (<200 cells/μl) and only about or below 25 mg/ml protein (7, 8, 9). Proteins detected in SF may originate from the surrounding tissues, such as lubricin—also known as proteoglycan 4—which plays a key role in the lubrication of the cartilage surface and is secreted by chondrocytes (10, 11). Yet, also plasma proteins are frequently detected in SF (12, 13, 14). The latter are thought to derive from the capillaries vascularizing the synovial tissue and to enter SF as an ultrafiltrate, which first penetrates the capillary endothelium and later the synovial lining (15, 16). Almost per definition, this filtration of plasma proteins from blood to SF may induce a bias, whereby the filtration of plasma proteins before they enter the SF may take place based on size, charge, or any other biochemical or biophysical properties of the proteins. In agreement, very early reports suggested that the extent of filtering from blood to SF depends on the molecular dimensions of the transported proteins (16, 17, 18, 19).

Pathological conditions, such as RA, however, imply drastic changes in the physiology of the joint, with severe consequences for the composition of SF (Fig. 1). Joint swelling in RA, for instance, reflects inflammation of the synovial tissue upon immune activation, which coincides with an expansion of the synovial lining and increases the permeability of the contained vasculature, enabling leukocytes to infiltrate the synovial compartment (1). Concomitantly, the cells forming the synovial lining are activated, resulting in the release of cytokines and proteases, such as matrix metalloproteinases (MMPs), which are involved in degrading the cartilage matrix (3). Increased numbers of cells, mostly neutrophils, also infiltrate SF (20) and the protein content in SF rises (7). In line, an increased permeability was reported for “rheumatoid” as compared with “healthy” joints (16, 17, 18).

In early reports, it has been reported that this increase in permeability is approximately six times for albumin but even more than 40 times for immunoglobulins (16). The studies underlying this estimation were, however, largely restricted in their depth, that is, investigated only a few selected plasma proteins or assessed immunoglobulins as a whole, neglecting the majority of proteins present in blood and potential differences in the abundance of individual antibody clones. Moreover, previous in-depth proteomics studies were limited to SF, thus, lacking information on paired plasma proteomes (12, 13, 21). As in-depth knowledge on the permeability of the synovial tissue is of great importance, for example, to judge the applicability of novel protein-based therapy formats and to assess the pharmacokinetics of systemically administered therapeutics, we here systematically revisited the filtration between blood and SF of RA patients suggested by Levick *et al.* in the 1980s and 1990s using advanced mass spectrometry (MS)–based proteomics methods.

**Fig 2:**
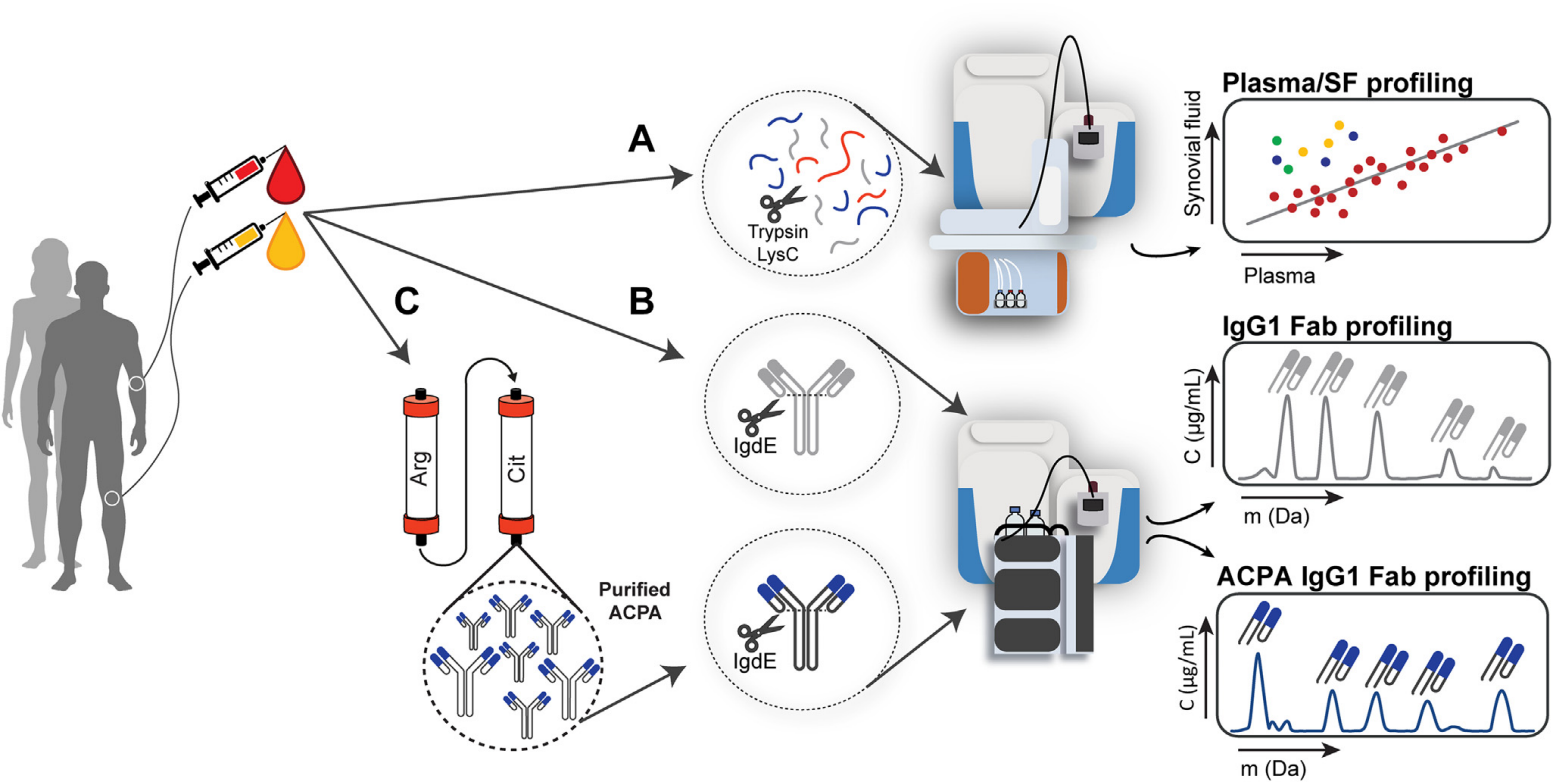
**Assessing the plasma and synovial fluid (SF) proteome at three molecular levels of depth.***A*, shotgun bottom–up proteomics analysis of paired plasma and SF samples with data-independent acquisition (DIA), resulting in correlation plots of plasma *versus* SF protein abundances. *B*, total IgG1 antibody repertoire analysis of paired plasma and SF samples using mass spectrometry–based Fab profiling. *C*, ACPA IgG1 autoantibody subrepertoire analysis of paired plasma and SF samples using autoantigen-specific Fab profiling. ACPA, anticitrullinated protein antibody; IgG1, immunoglobulin G1.

We hypothesized that protein concentrations in plasma and SF of the same donor would reveal biases in certain proteins being relatively more or less abundant in either fluid possibly based on their size or charge if filtering takes place. We considered that the distribution of plasma proteins between both compartments can provide insights into the biodistribution of protein-based therapeutics administered to RA patients to treat disease. To this end, we analyzed the plasma and SF proteome of patients diagnosed with RA at three distinct molecular levels of depth (Fig. 2): First, we analyzed the “total” plasma and SF proteome qualitatively and quantitatively using robust data-independent acquisition (DIA), compared the plasma and SF proteome within each patient and between patients, and correlated the abundances of over 300 different proteins detected in both fluids. Next, we assessed the total immunoglobulin G1 (IgG1) antibody repertoires at molecular resolution using MS-based Fab profiling (22), comparing the plasma and SF IgG1 repertoires within each patient and between the patients; and eventually analyzed and characterized the anticitrullinated protein antibody (ACPA) IgG1 autoantibody subrepertoires using our recently introduced autoantigen-specific Fab profiling approach (23). Interestingly, at all these levels, plasma proteins and antibody repertoires detected in SF highly resembled those in plasma of the same patient both qualitatively and quantitatively. However, proteins that were exclusively detected or enriched in SF were indicative for the local (immune) processes destructive to cartilage. We therefore conclude that plasma proteins—and concomitantly systemically administered (protein-based) therapeutics—directly pass into SF of swollen joints of RA patients without substantial bias; we thereby exemplify the use of in-depth multilayer proteomics analyses to revisit former concepts underlying RA pathology as well as to monitor the local (immune) processes; and propose that pharmacokinetic analyses of systemically administered protein-based therapeutics in blood are directly relevant to the synovial compartment and that entering of plasma into SF may contribute to diminishing the unique properties of SF, such as lubrication, eventually impairing joint functioning.

## Experimental Procedures

### Experimental Design and Statistical Rationale

For the study, paired blood and SF samples were obtained from nine patients, including three male and six female patients in the age of 44 to 73 years (Table 1). All patients were diagnosed with RA prior to sampling and met the 2010 American College of Rheumatology/European Alliance of Associations for Rheumatology criteria for RA at the time of diagnosis. All patients visited the outpatient clinic of the Rheumatology Department at the Leiden University Medical Center at the time of sampling and gave written informed consent before sample donation. Detailed characteristics of the study cohort are summarized in Table 1. The permission for conduct of the study was approved by the Ethical Review Board of the Leiden University Medical Center (protocols P13.171 and P17.151) abiding by the Declaration of Helsinki principles.

**Table 1:** Characteristics of the patient cohort studied

Patient	Age (years)	Sex	Duration (years)	DAS28	ESR	Treatment at time of sampling
01	58	Male	1	2.79	6	Methotrexate 25 mg q.w.
02	57	Male	26	4.04	48	Methotrexate 20 mg q.w., sulfasalazine 3 g q.d., hydroxychloroquine 400 mg q.d., azathioprine 100 mg q.d.
03	62	Female	1	5.09	99	Methotrexate 15 mg q.w., prednisolone 2.5 mg q.d.
04	54	Male	0	3.01	9	None
05	62	Female	9	4.05	36	Abatacept 125 mg s.c. q.w., leflunomide 20 mg q.d.
06	73	Female	20	5.71	50	Methotrexate 10 mg q.w.
07	49	Female	19	4.24	28	Methotrexate 20 mg q.w., adalimumab 40 mg s.c. q.o.w.
08	67	Female	24	3.12	2	Tocilizumab 162 mg s.c. q.w., prednisolone 5 mg q.d.
09	44	Female	33	5.21	86	Certolizumab pegol 200 mg q.o.w.

Samples were collected prospectively over a period of 8 years. All patients were diagnosed with ACPA-positive RA prior to sampling. Patient 05 was additionally diagnosed with Crohn’s disease. Disease duration is given as time span between the year of diagnosis and the year of sampling.

DAS28, disease activity score 28 with erythrocyte sedimentation rate; ESR, erythrocyte sedimentation rate; q.d., daily; q.w., weekly (once a week); q.o.w.: every other week; s.c., subcutaneous.

### Collection and Preparation of Plasma and SF

SF was obtained by therapeutic arthrocentesis and separated from synovial cells by centrifugation for 10 min at 2000 rpm and 20 °C. To reduce the viscosity, SF was mixed 1:11 with 1 mg/ml sterile-filtered hyaluronidase (10 volume-equivalents SF:one volume equivalent hyaluronidase) prior or after separation from synovial cells. Paired plasma was collected from peripheral blood taken from the peripheral vein shortly after arthrocentesis and prepared either through Ficoll–Paque-based isolation of peripheral blood mononuclear cells after dilution of whole blood with PBS (patients 01, 02, 03, and 09) or after centrifugation of whole blood for 10 min at 2000 rpm and 20 °C (patients 04–08). All plasma and SF samples were stored at −20 °C until further use.

### Sample Preparation for Proteomics

8 of 18 samples (four plasmas and four SFs) were used to determine the protein concentration with a Bradford protocol using Bio-Rad Protein Assay Dye Reagent Concentrate (Bio-Rad; catalog no.: 5000006) according to the manufacturer’s instructions. The calibration curve ranged from 0 to 5 μg and was performed in duplicates. Plasma samples were diluted 50 or 100 times, SF samples were diluted 10 or 20 times. All samples were analyzed in duplicate. Absorbance was measured with a Multiskan GO (Thermo Fisher Scientific) at a wavelength of 595 nm. The calibration curve had an *R*^2^ of 0.97. Subsequently, approximately 10 μg of protein was denatured, reduced, and alkylated by adding 100 μl of 150 mM Tris, 5 mM Tris(2-carboxyethyl) phosphine, 30 mM chloroacetamide, and 1% sodium deoxycholate of pH 8.5. Then, 100 ng of LysC (Wako) and 100 ng of trypsin (Promega) were added, and the sample was incubated and digested overnight at 37 °C. The samples were then acidified by adding formic acid (FA) to a concentration of 0.5% prior to solid-phase extraction sample clean-up, causing sodium deoxycholate to precipitate. Solid-phase extraction clean-up was performed on an Oasis HBL U-elution plate. After the clean-up, the samples were dried with a vacuum centrifuge and reconstituted in 2% FA prior to analysis. Approximately 1 μg of reconstituted peptides were injected in the liquid chromatography–coupled mass spectrometer.

### LC–MS/MS DIA for Proteomics

All spectra were acquired on an Orbitrap Exploris 480 mass spectrometer (Thermo Fisher Scientific) operated in data-independent mode coupled to an Evosep One liquid chromatography system, using the Endurance column (EV1106) at 30SPD method (44 min gradient). The samples were loaded on Evotip C18 disposable trap columns (EV2018). The DIA method consisted of MS1 scans at 60,000 resolution, and scan ranges between 375 and 1600, followed by 50 scan windows with 12 *m/z* and one window overlap in the high collision dissociation MS/MS. The resolution on the Orbitrap was set to 15,000 at 200 *m/z* with precursor mass range of 400 to 1000 *m/z*. Proteome samples were eluted over a linear gradient of a dual-buffer setup with buffer A (0.1% FA) and buffer B (80% acetonitrile and 0.1% FA) ranging from 9 to 44% B over 65 min, 44 to 99% B for 3 min, and maintained at 95% B for the final 5 min with a flow rate of 300 nl/min. DIA runs consisted of an MS1 scan at 60,000 resolution at *m/z* 200 followed by 30 sequential quadrupole isolation windows of 20 *m/z* for high collision dissociation MS/MS with detection of fragment ions in the Orbitrap at 30,000 resolution at *m/z* 200. The *m/z* range covered was 400 to 1000, and the automatic gain control was set to 100% for MS and 1000% for MS/MS. The injection time was set to “custom” for MS and “auto” for MS/MS scans.

### Raw Data Processing and Statistical Analysis of Proteomics

All samples were analyzed in one run using DIA-NN’s software (version 1.8.1) (24) in library-free mode. Trypsin was selected as the protease, and two missed cleavages were tolerated. Cysteine carbamidomethylation and methionine oxidation were allowed. The mass accuracy was set to 20 and the MS1 accuracy to 10. The unrelated runs, isotopologs, match-between-runs, and no shared spectra options were enabled, whereas the heuristic inference was disabled. The protein inference was set to Genes, and the IDs, RT, and IM profiling was used for the library generation. The false discovery rate was at the default 1%. The reviewed UniProt human protein database (canonical and isoforms) was used, with ∼42,000 entries (Release number 2023_09) together with a manually curated contaminants database of around 150 entries. The main report of DIA-NN was filtered from the contaminants and used for further analysis. As it is well established in plasma proteomics to use the intensity of a protein in label-free quantification (LFQ) as a proxy for protein concentrations, MaxLFQ values were selected to determine protein abundances and calculated together with the number of unique peptides per protein in each sample using the *diann_maxlfq* function of the DIAgui package (https://github.com/mgerault/DIAgui). The Q.Value, Lib.Q.Value, and Lib.PG.Q.Value were set to 1%. Only proteins with unique peptides identified in at least six of nine plasma or SF samples were included for the MaxLFQ calculation. NA values were imputed with the lowest LFQ value of 50783.6. All downstream analyses were carried out in R software (25).

To crosscorrelate proteomes, the identified proteins were further filtered using the following criteria: I. proteins describing the variable domain of immunoglobulins were removed; II. The LFQ intensity of a protein had to be above 5e5. All remaining proteins, their detected abundance in each of the 18 samples analyzed as well as the averaged plasma proteome (averaged over nine plasma samples), and the averaged SF proteome (averaged over nine SF samples) were used for crosscorrelation. An overview of all proteins identified prior to and after filtering as well as their abundances are provided in Supplemental Table S1, *A* and *B*, respectively.

### Protein Categorization

All identified proteins from Supplemental Table S1*B* were further divided into seven distinct categories based on their function and/or cellular origin, namely plasma proteins, cartilage proteins, proteins related to neutrophils, erythrocytes, platelets, or lesser defined generic cells. The erythrocyte- and platelet-related proteins were based on the quality control tool of the plasma proteome developed by Geyer *et al.* (26). The remaining categories were derived from available references and Gene Ontology annotations. Proteins that could not unambiguously be classified in either of these categories (or belonged to multiple categories) were labeled as noncategorized proteins. It should be noted that nearly all these noncategorized proteins were detected exclusively in the SF and often of lower abundance.

### Purification of ACPA From Plasma and SF

ACPA was purified from plasma and SF of patients 01, 02, 04, and 08 using a tandem purification approach as described previously (23). Briefly, HiTrap Streptavidin HP columns (GE Healthcare/Cytiva) conjugated with CArgP2 (the nonmodified version of cyclic citrullinated peptide 2 [CCP2]) and CCP2 were coupled in line to an ÄKTA pure purification system (GE Healthcare) and equilibrated with PBS. Plasma and SF were sterile filtered, and respective volumes were manually injected into a 5 ml sample loop and applied to tandem purification at a flow rate of 0.5 ml/min. After complete sample application, the sample loop and the columns were washed with PBS, and ACPA was eluted from the CCP2 column with glycine–HCl, pH 2.5 at a flow rate of 0.5 ml/min. ACPA was collected in fractions of 0.2 ml, and up to eight elution fractions starting from the beginning of the elution peak were pooled and desalted immediately after fractionation using 5 ml Zeba spin desalting columns (Thermo Fisher Scientific) according to the manufacturer’s instructions. Successful ACPA purification was monitored by enzyme-linked immunosorbent assays, and desalted ACPA was stored at 4 °C until IgG capturing. Volumes of paired plasma and SF applied to ACPA purification were determined and adjusted based on ACPA IgG levels in either fluid to exclude biases as a consequence of varying antibody levels.

### IgG Capturing and Fab Generation

The procedure for capturing IgG from purified ACPA and generating IgG1 Fab fragments followed the same procedure as previously described (23). In short, a Pierce Spin Column with 20 μl CaptureSelect FcXL affinity matrix slurry (Thermo Fisher Scientific) was washed three times with 150 mM phosphate buffer (PB). After washing, the affinity matrix was incubated with the purified ACPA samples, together with 100 ng of an internal standard mix (1:1 mix of the recombinant monoclonal antibodies trastuzumab:alemtuzumab) and 1% milk (Fantomalt; Nutricia) in fractions of up to 750 μl while rotating head-over-head at room temperature for each 1 h. Unbound fractions were collected by centrifugation, and after applying the entire ACPA sample, bound ACPA IgG was washed four times with PB. Subsequently, ACPA IgG1 Fab fragments were generated by selectively cleaving captured IgG1 using 50 units of the IgG1-specific immunoglobulin-degrading enzyme (FabALACTICA; Genovis) in 50 μl PB by incubating for at least 16 h at 37 °C on a thermal shaker (22). The resulting ACPA IgG1 Fab fragments were collected by centrifugation for 1 min at 500*g*. To capture IgG from plasma and SF, CaptureSelect FcXL affinity matrix was prepared as described previously. After washing, the matrix was resuspended in 150 μl PB with 400 ng of the internal monoclonal antibody (mAb) standard mix. A plasma or SF volume containing an estimated 50 μg IgG1 was added and incubated with the affinity matrix for 1 h shaking at room temperature. After four PB washes, IgG1 Fab fragments were generated as described previously.

### LC–MS-Based Fab Profiling

To analyze the released intact Fab fragments, a reversed-phase LC–MS method was employed, as detailed earlier (22, 23). In short, collected Fab fragments were separated using a Vanquish Flex UHPLC system with a 1 × 150 mm MAbPac Reversed Phase HPLC column, coupled to an Orbitrap Exploris 480 MS with BioPharma option. The IgG1 Fab fragments were chromatographically separated over a 62-min gradient, at a flow rate of 150 μl/min and with both the column preheater and the analytical column chamber heated to 80 °C. Mobile phases A (0.1% HCOOH in MilliQ water) and B (0.1% HCOOH in CH_3_CN) were used for gradient elution. MS data were collected in intact protein and low-pressure mode. Spray voltage was set at 3.5 kV, ion transfer tube temperature at 350 °C, vaporizer temperature at 100 °C, sheath gas flow at 15 arbitrary units, auxiliary gas flow at 5 arbitrary units, and source-induced dissociation at 15 V. Spectra were recorded with a resolution setting of 7500 (@*m/z* 200) in MS1, covering a range of 500 to 4000 *m/z*, with an automatic gain control target of 300% and a maximum injection time of 50 ms. For each scan, 5 microscans were recorded.

### Fab Profiling Data Analysis

For LC–MS analysis, BioPharmaFinder 3.2 (Thermo Fisher Scientific) was used to retrieve retention time and mass (in Dalton) from RAW files, following the same method as previously described (22, 23). In short, deconvolution was performed using the ReSpect algorithm (Thermo Fisher Scientific) using 0.1 min sliding windows, 25% offset, a merge tolerance of 30 ppm, and a noise rejection set at 95%. The output range was put between 10,000 and 100,000 Da, targeting a mass of 48.0 kDa and a 30 ppm mass tolerance, considering charge states between 10 and 60 and using the Intact Protein Peak model. Further analysis was conducted using in-house Python scripts, as detailed in previous work (22, 23). In short, masses were recalculated using an intensity weighted mean, focusing on the most intense peak comprising 90% of the total intensity. Fab fragments within 45.0 and 56.2 kDa with the most intense charge state above 1000 *m/z* and a BioPharmaFinder score ≥40 were identified to be Fab fragments of IgG1 antibodies. Identical Fab fragments were determined within a mass and retention time window of three times the standard deviation of the respective value observed for the mAb standard and some most intense Fab molecules. Concentrations of detected Fab molecules were determined by normalizing their sum intensity to the averaged sum intensity of the internal mAb standard. Corrections were made for the plasma or SF volume applied to ACPA purification or total IgG1 Fab profiling. The mAb standard was subtracted from the list of Fab fragments detected, and the remaining Fab fragments were considered unique Fab molecules based on their unique pair of mass and retention time. Overlapping Fab profiles were quantified, and the degree of overlap is presented as a percentage in the overlap heatmaps.

## Results

### Protein Composition of Paired Plasma and SF From RA Patients

To revisit the suggested filtration of plasma proteins from blood to SF of RA patients, we first determined the protein composition within the two body fluids by analyzing the total proteome of nine paired plasma and SF samples of patients diagnosed with RA that underwent therapeutic arthrocentesis because of joint swelling (n = 18). Using a robust DIA approach as described earlier (27, 28), we identified 481 unique proteins (551 proteins including those with shared peptides) across all 18 samples. Most of the detected proteins (on average, 33%) represented plasma proteins, whereas additional proteins were detected, related largely to cartilage, and proteins originating from cells such as neutrophils, erythrocytes, and/or platelets.

### Cartilage Proteins and Cellular Proteins Derived From Neutrophils and Other Cells are Distinctively Enriched and/or Identified in SF

**Fig 3:**
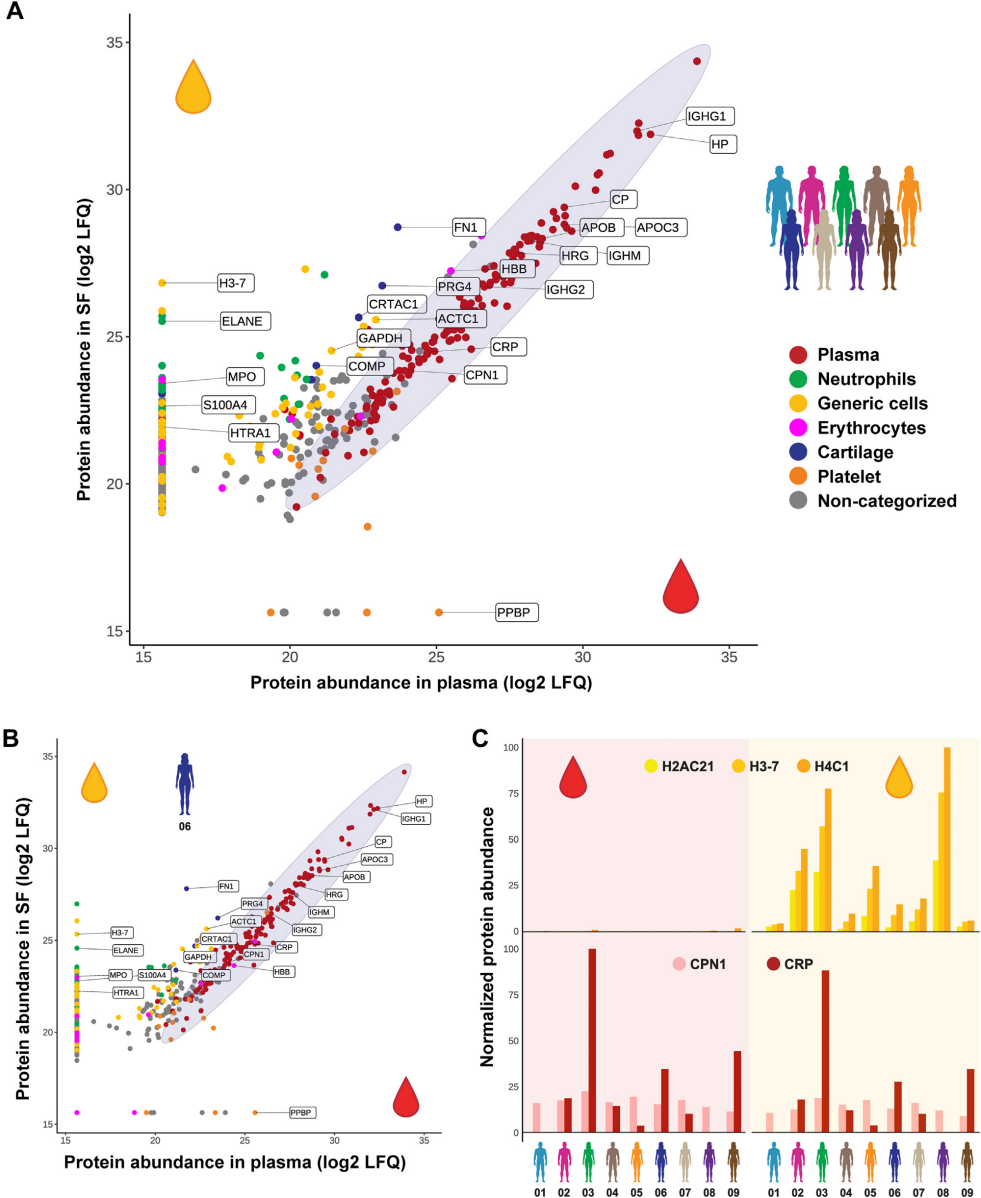
**Proteomics-based characterization of paired plasma and synovial fluid (SF).** Quantitative overview of the relative abundance of the 481 unique proteins comparing their abundance (as estimated based on LFQ values) in plasma *versus* SF of nine rheumatoid arthritis (RA) patients. Undisputable plasma proteins are annotated in *red*. Proteins related to cartilage/chondrocytes, erythrocytes, neutrophils, platelets, and generic cells are annotated in *blue*, *pink*, *green*, *orange*, and *yellow*, respectively. Proteins that could not be easily classified are indicated in *gray*. *A*, abundances as averaged over all nine patients. *B*, illustrative abundances obtained for patient 06. Respective abundances for all other individual patients are provided in Supplemental Fig. S1. While plasma proteins correlate in abundance very well between plasma and SF (*i.e.*, within the *ellipse* provided to guide the eye) in these RA patients, the other protein categories are mostly enriched in SF. *C*, normalized protein abundance of histones H2AC21, H3–7, and H4C1 (*top*), present nearly uniquely in SF, and CRP and CPN1 (*bottom*) in all analyzed samples. CRP levels vary among the patients but correlate well between the plasma and SF of the matching donor. The LFQ values were normalized by subtracting the minimum from the original LFQ value and dividing by the difference of maximum and minimum LFQ. CRP, C-reactive protein; LFQ, label-free quantitation.

When correlating the abundance of each detected protein in plasma and SF averaged over all patients (Fig. 3*A*) or for each donor individually (Figure 3*B* and Supplemental Fig. S1), paired proteomes show a strikingly good correlation, reflected by a Pearson correlation of 0.96 and 0.95, respectively. Nonetheless, several proteins were substantially more abundant or solely detected in SF.

The latter included, as expected, cartilage proteins, including cartilage oligomeric matrix protein (COMP), cartilage acidic protein 1 (CRTAC1), lubricin (PRG4), the procollagen C-endopeptidase enhancer (PCOLCE), fibronectin (FN1), and the aggrecan core protein (ACAN or PGCA)—a proteoglycan and major component of extracellular matrix of cartilaginous tissues. The detected overall abundances of cartilage proteins thereby differed between patients: While relatively low amounts of cartilage-originating proteins were detected in the SF of patients 03, 04, and 06, we detected relatively high amounts of those proteins in SF of patients 01, 02, 05, 07, 08, and 09 (Supplemental Fig. S1).

Next to cartilage proteins, several proteins originating from cell nuclei (notably histones, *e.g.*, H2AC21, H3–7, and H4C1) and cellular cytoskeleton were enriched or solely detected in SF. These proteins were particularly enriched in SF of patients 02, 03, 05, and 08, whereas patients 01 and 09 had the lowest number of histones detected in their SF (Fig. 3*C*). Alike patterns were also observed for various actin proteins (*e.g.*, ACTC1, ARPC2, ACTR3) but also the cellular household protein GAPDH (Supplemental Fig. S2*A*).

Other proteins enriched or solely detected in SF originate from neutrophils. Some of the most abundant of these proteins are neutrophil elastase (ELANE), myeloperoxidase (MPO), enolase-1 (ENO1), cathepsin G (CTSG), α-defensin (DEFA1), myeloblastin (PRTN3), S100-A8, and S100-A9, which are all known to be enriched in neutrophils (29). Moreover, several MMPs were uniquely detected in SF, with MMP-1, MMP-3, and MMP-9 being the most abundant in the studied cohort (Supplemental Fig. S2*B*).

### All Major Plasma Proteins Are Present in SF in Interconnected Abundance

In all 18 analyzed plasma and SF samples, we detected constantly more than 150 proteins annotated as “classical” plasma proteins. In contrast to cartilage proteins and cellular proteins derived from neutrophils or other cells, our analyses showed a remarkably different picture for authentic plasma proteins. Although these plasma proteins span collectively an order of difference in plasma abundance of around 10^4^, the abundance of any protein in plasma correlated well with that in SF of the same donor. This includes some of the most well-known and abundant plasma proteins, such as albumin and several plasma glycoproteins, such as haptoglobin, histidine-rich glycoprotein, and ceruloplasmin. For some plasma proteins, interdonor variability in concentration is known to be higher than for others. To illustrate this, the determined concentrations of carboxypeptidase N (CPN1) (Fig. 3*C*; *light red bars*) do not vary substantially between the plasma samples of all nine patients and are also quite alike those determined in the nine SF samples. Conversely, C-reactive protein, a well-known marker for inflammation, was not detected in patients 01 and 08 but very high in for instance patients 03, 06, and 09 (Fig. 3*C*; *dark red bars*), both in plasma and SF. In line, patients 03, 06, and 09 also showed the highest disease activity as represented by DAS28 scores (Table 1).

While 165 of 481 proteins were solely detected or enriched in SF, only a very limited number of proteins was solely detected or enriched in plasma, namely the platelet basic protein, platelet factor 4, and thrombospondin-1. These proteins likely originate from platelets and have been reported as potential artifacts in the preparation of plasma, being strong indicators of coagulation and acknowledged causes of preanalytical variation (26, 30, 31).

### The Abundance of Plasma Proteins in SF is Independent of Their Size

Early reports suggested that the extent of filtering from blood to SF depends on the molecular characteristics of the transported proteins. Considering the overall high correlation of the >150 detected plasma proteins, we therefore wondered whether the observed correlation is still biased by, for instance, the molecular weight of the respective protein. To this end, we selected 19 proteins and/or protein complexes of relatively low (∼40–100 kDa), medium (∼110–500 kDa), and high (∼510–1000 kDa) molecular weight and assessed their abundances in paired plasma and SF. The molecular weight of the monomeric or multimeric complexes was based on UniProt or previous literature and described in Supplemental Table S2. Remarkably, both lower, medium, and higher molecular weight proteins and protein assemblies from ∼40 kDa to 1000 kDa displayed an exceptional correlation in protein abundance in plasma and SF of the matched donor (Supplemental Fig. S3). Our data thus suggest that plasma proteins enter SF of swollen joints of RA patients without size-based constraints.

### IgG1 Antibodies are Shared Between Plasma and SF in Interconnected Abundance

**Fig 4:**
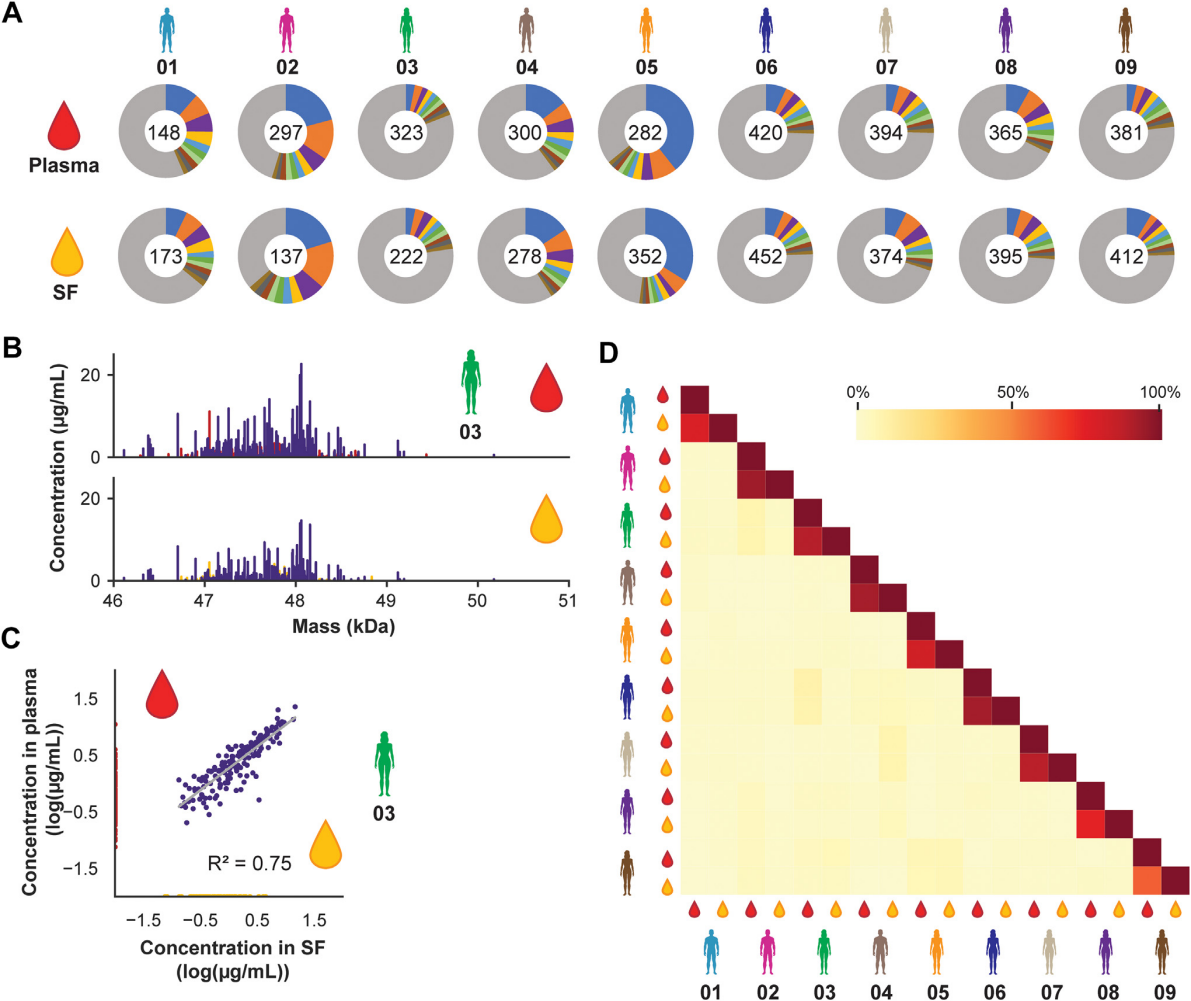
**Characterization of paired plasma and synovial fluid (SF) IgG1 repertoires using LC–MS-based Fab profiling.***A*, number and relative abundance of unique IgG1 Fab molecules detected in plasma (*top row*) and SF (*bottom row*) of all n = 9 rheumatoid arthritis (RA) patients studied. Unique Fab molecules were defined based on their unique pair of mass and retention time. The number in the center of each donut plot depicts the total number of Fab molecules detected. The contribution of each of the 10 most abundant Fab molecules is provided by color, with the most abundant Fab molecule in *blue*. Illustratively, for patient 05, the most abundant Fab molecule contributes ∼40% to the detected IgG1 repertoire; for patient 03, this is just 3%. Note that qualitatively and quantitatively the color palettes in the matched plasma–SF pie charts are very much alike. *B* and *C*, exemplary IgG1 Fab mass profile obtained for plasma (*top*) and SF (*bottom*) of patient 03 (*B*) and correlation of the detected IgG1 Fab concentrations between both fluids (*C*), further displaying the high similarity between paired plasma and SF repertoires. Fab molecules shared between both fluids are depicted in *purple*, Fab molecules uniquely detected in plasma or SF are depicted in *red* and *yellow*, respectively. The correlation of IgG1 Fab molecules detected in both plasma and SF was determined based on the logarithmic concentration of each Fab molecule using linear regression. The resulting regression line is shown in *gray* (*R*^2^ = 0.75; *p* < 0.001). If a Fab molecule was not detected in one of the body fluids, a concentration of 0.01 μg/ml was imputed. *D*, intensity-based correlation matrix depicting the correlation of the measured IgG1 repertoires within and between patients. A high correlation is observed between plasma and SF repertoires within each RA patient, whereas weak to no correlation is observed between repertoires from different RA patients. IgG1, immunoglobulin G1.

Immunoglobulins form a major part of the plasma proteome (∼30% in protein concentration) and can also enter the SF as indicated previously (Fig. 3). In the proteomics study, we evaluated total levels of, for example, IgG1 and IgM. Nevertheless, each immunoglobulin isotype entails hundreds to thousands of individual clones that are distinctive in their abundance (22, 23, 32). To further explore the comparability between plasma and SF, we used our previously introduced LC–MS-based IgG1 Fab profiling approach. This method allows us to resolve the molecular diversity of the IgG1 repertoires in both plasma and SF and to quantify the concentration of the most abundant antibody clones present (22). Using IgG1 Fab profiling, we were able to distinguish a few hundred (∼140–450 per sample) unique Fab molecules in each of the total IgG1 Fab repertoires, both for plasma and SF. For some patients, for example, patient 03, the detected IgG1 Fab molecules ranged in concentrations between 0.1 and 25 μg/ml, whereas for some other patients, for example, patients 02 and 05, we detected Fab molecules with concentrations exceeding 100 μg/ml (Supplemental Fig. S4). In all patients, the 10 most abundant IgG1 Fab molecules in paired SF and plasma samples displayed alike relative abundances (Fig. 4*A*). Beyond these top 10, also the mass distribution and abundances of the remaining Fab molecules detected in plasma and SF showed to be highly similar (Fig. 4*B*). This we again visualized by correlating the concentrations of the IgG1 Fab molecules in plasma and SF (Fig. 4*C*) observing again a remarkably high correlation (R^2^ = 0.75, *p* < 0.001). Finally, a correlation matrix that compares the IgG1 repertoires within and between patients, using a concentration-dependent correlation analysis (22), shows no strong correlation for IgG1 repertoires from different patients, in line with our previous work (22). Yet, this analysis shows a very strong correlation between the IgG1 repertoires in plasma and SF from the same RA patient (Fig. 4*D*). Our findings at these two levels of molecular entities (total proteome and IgG1 repertoires) thus demonstrate that plasma and SF correlate very well not only at the total IgG, IgA, and IgM levels as detected by proteomics (Fig. 3) but also when comparing the IgG1 repertoires in plasma with those in SF in paired samples of the same donor (Fig. 4).

### Systemically Administered IgG1-Based Therapeutics Can be Traced Back in Plasma and SF

Several patients in our cohort received IgG1-based mAb therapy (Table 1). We previously showed that we can trace such systemically administered therapeutic mAbs back in the clonal LC–MS Fab profiles based on their distinctive mass and retention time (33). Patient 07 was treated with adalimumab, an anti-tumor necrosis factor-α IgG1-based mAb. Previous data on the pharmacokinetics of adalimumab in combination with methotrexate imply an expected plasma concentration of ∼8 to 9 μg/ml when administered at a dose of 40 mg, as given to this patient (34). This aligns nicely with the here observed concentration of about 9.0 μg/ml. Interestingly, adalimumab was also detected in the SF of patient 07 at an alike, albeit somewhat lower concentration of 3.2 μg/ml. Patient 08 received tocilizumab, an anti-IL-6R IgG1-based mAb. In this patient’s plasma, we detected tocilizumab at a concentration of 18 μg/ml, which is on the lower end of the range reported in the literature for a 162 mg injection. This lower concentration could be attributed to the timing of sampling. In addition, the concomitant use of prednisone, which was not accounted for in previous studies (35), might influence the tocilizumab concentrations. Again, tocilizumab could also be detected in the SF of, exclusively, this patient. Thus, also systemically administered therapeutics enter the plasma and SF of RA patients to similar levels of concentrations.

### Also, ACPA IgG1 Subrepertoires of Paired Plasma and SF Display Interconnected Abundances

**Fig 5:**
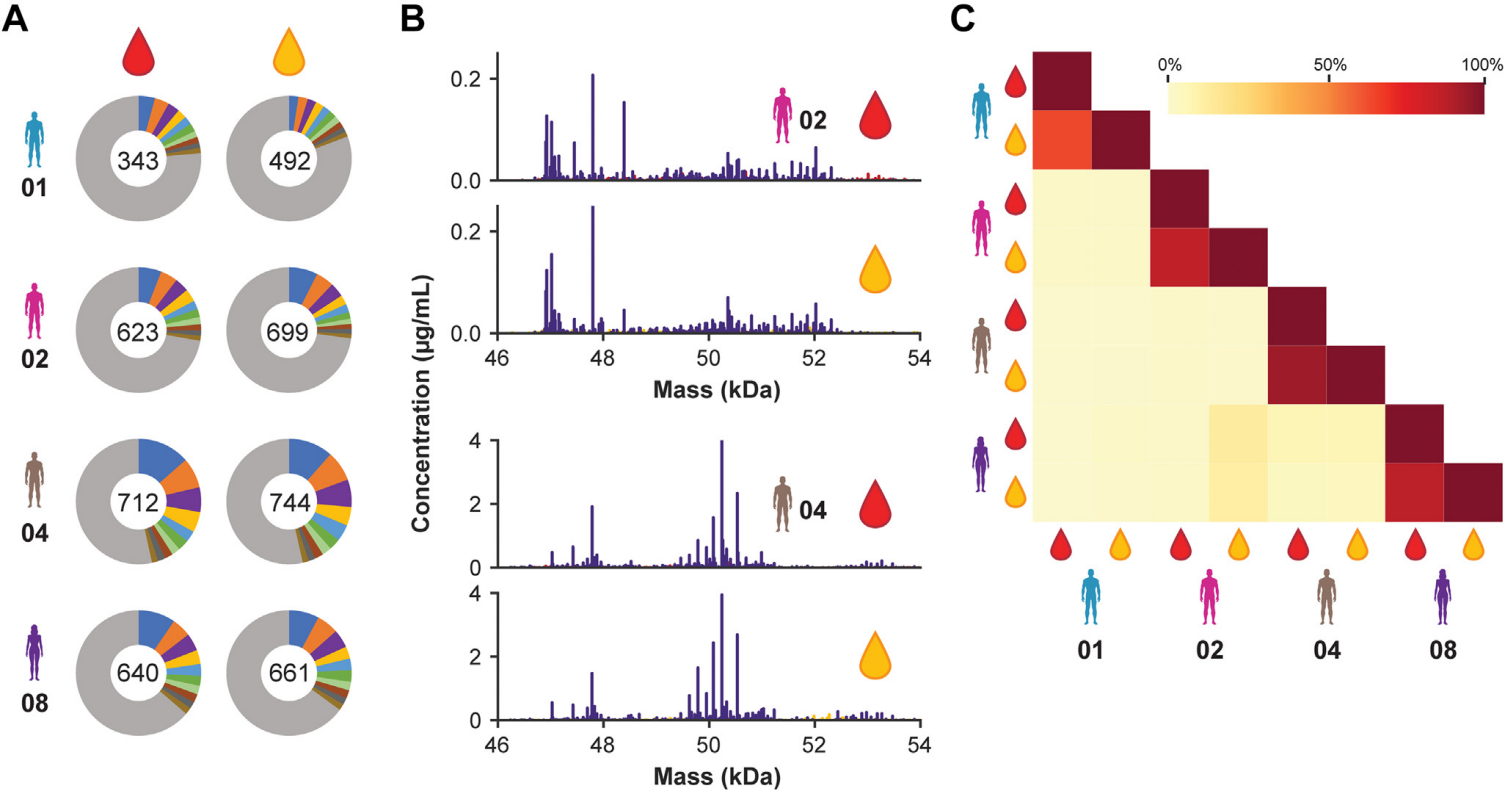
**Characterization of paired plasma and synovial fluid (SF) ACPA IgG1 repertoires using ACPA IgG1 Fab profiling.***A*, number and relative abundance of unique ACPA IgG1 Fab molecules detected in plasma (*left column*) and SF (*right column*) of n = 4 rheumatoid arthritis (RA) patients. Unique Fab molecules were defined based on their unique pair of mass and retention time. The number in the center of each donut plot depicts the total number of Fab molecules detected. The contribution of each of the 10 most abundant Fab molecules is provided by color, with the most abundant Fab molecule in *blue*. *B*, representative ACPA IgG1 Fab profiles obtained for plasma and SF of patients 02 and 04. Fab molecules shared between both fluids are depicted in *purple*, Fab molecules uniquely detected in plasma or SF are depicted in *red* and *yellow*, respectively. Notably, because of ACPA Fab glycosylation, the mass of many ACPA Fab molecules is distinctively higher (*i.e.*, >49 kDa). *C*, intensity-based correlation matrix comparing plasma and SF ACPA IgG1 repertoires within and between patients, revealing the high correlation of ACPA IgG1 repertoires within each RA patient and a low correlation of ACPA IgG1 repertoires between RA patients. The ACPA Fab profiles obtained for plasma and SF of patients 01 and 08 are shown in Supplemental Fig. S5. ACPA, anticitrullinated protein antibody; IgG1, immunoglobulin G1.

Our work described previously demonstrates that both the total proteome and the total IgG1 repertoires of plasma and SF from a single RA patient closely resemble each other both qualitatively and quantitatively. These findings suggest that there is no distinctive barrier between plasma and SF in swollen joints of RA patients. However, our analysis of the total IgG1 repertoires focuses on the several hundred most abundant IgG1 antibody clones. There may, however, still be differences in abundance when examining the IgG1 repertoire more closely, particularly when focusing on RA-related autoantibodies, such as ACPA. Previously, we and others already estimated that the ACPA subrepertoire constitutes approximately 1% of the total IgG1 repertoire (36). Using our previously introduced ACPA-focused approach of Fab profiling (23), we next selectively purified the ACPA repertoire of both plasma and SF of four of the nine patients, patients 01, 02, 04, and 08. This method yielded highly informative ACPA IgG1 repertoires, each containing hundreds of unique ACPA IgG1 Fab molecules. Our findings here are consistent with our previous work (limited to plasma), which showed that ACPA subrepertoires are unique to each patient. However, strikingly, when comparing the ACPA IgG1 repertoires between paired plasma and SF, we found that they are highly similar as evidenced by the comparable abundances of the most abundant ACPA Fab molecules (Fig. 5*A*) and the highly similar mass distributions (Fig. 5*B* and Supplemental Fig. S5), resulting in a strong correlation of the ACPA subrepertoires in plasma and SF for each patient as shown in the correlation matrix (Fig. 5*C*). Thus, the IgG1 repertoires are highly similar between plasma and SF of RA patients, both at the total level (Fig. 4) and the ACPA-specific level (Fig. 5).

## Discussion

SF typically serves as joint lubricant and nutrient source for the nonvascularized articular cartilage (5). Nevertheless, also plasma proteins are frequently detected, which are thought to derive from the capillaries vascularizing the synovial tissue. Already more than 50 years ago, studies suggested that the passage of plasma proteins into SF may depend on the molecular dimensions of the respective protein (16, 17, 18, 19). Such a bias of proteins moving from blood to SF is of great relevance, especially with respect to the biodistribution of systemically delivered therapeutics. The availability of mAb treatments for the therapy of RA has transformed the treatment of RA within the past decades (37, 38). However, with the recent advent of multispecific antibodies, antibody derivatives, and larger polymer scaffolds (39, 40, 41), even more complex therapeutics are conceivable. These may offer enhanced selectivity or multivalency, but they may also be differentially affected by a potential filtration bias from blood to SF. As previous studies investigating the permeability of the synovium for plasma proteins were largely restricted to a few selected proteins (18, 19), we here systematically revisited the filtration between blood and SF in swollen joints of RA patients using a multitiered proteomics approach. We hypothesized that protein concentrations in plasma and SF from the same donor would show biases in the relative abundance of certain proteins in each fluid, potentially influenced by their size or charge if filtering occurs. We also anticipated that such analyses might shed light on how protein-based therapeutics are distributed between plasma and SF in RA patients.

Using robust DIA, we first characterized the protein composition of paired plasma and SF and identified and quantified ∼481 unique proteins. In line with a previous proteomic study of healthy porcine SF, the largest group of proteins detected in SF can be considered as “classical” plasma proteins, whereas a smaller fraction of proteins was linked to cartilage (21). Additional proteins detected could be classified as generic cellular proteins and as proteins related to neutrophils, erythrocytes, or platelets.

In line with a previous report (42), fibronectin, which is known to play a role in cartilage regeneration (43), was consistently enriched in SF when compared with plasma of the same RA patient, suggesting a local production of fibronectin related to the chronic inflammatory process. Other cartilage proteins, such as cartilage oligomeric matrix protein, are often considered as indicator of cartilage turnover (44, 45). Besides, MMPs and the serine protease HTRA1, both solely detected in SF, are known for their roles in cartilage degradation (46, 47). Remarkably, the abundance of generic cellular proteins, such as histones, actin proteins, and GAPDH, showed to associate with each other but to vary between individuals and may thus serve as an indicator of the extent of (inflammation-induced) cell death within the respective synovial joint. Neutrophils, the major cell population infiltrating into RA SF upon joint inflammation, are known to release proteins contained in their granules through the process of NET formation (20, 48). The detection of such neutrophil-derived proteins, including myeloperoxidase, neutrophil elastase, cathepsin G, and proteinase 3, in SF consequently represents the local (proinflammatory) immune response. The enhanced presence of proteases in SF might thereby also explain why constant domains obtained from IgG molecules of SF appear differentially modified as compared with respective constant domains of plasma of the same donor (49).

In contrast to the proteins reflecting local (immune) processes, which are consequently enriched or solely detected in SF, intriguingly, hardly any protein was solely detected in plasma. Even more, the abundance of genuine plasma proteins in plasma highly correlated with their abundance in SF. This correlation is particularly remarkable as the detected plasma proteins differed in their abundance in plasma by about 10^4^ and ranged in molecular weight between ∼10 kDa and 1000 kDa. Although minor deviations, for instance, as a consequence of modification, local consumption, or local binding to cartilage or other components of SF or the joint cavity may impact the abundance of individual proteins in either of both fluids, our data thus provide direct evidence that (i) seemingly all plasma proteins enter SF of the same donor and (ii) they enter SF to a similar extent.

In addition, the abundance of the major immunoglobulins correlated between plasma and SF similarly to the remaining plasma proteins. This observation is particularly striking as several studies have reported that antibodies can be produced locally in the synovial tissue and SF of RA patients (50, 51, 52, 53) or have reported a marked enrichment of IgG and IgM as compared with other plasma proteins (16, 18). A study, already originating from more than 50 years ago, even reported that about 12 to 24% of the IgG present in SF of five RA patients was derived by local production (54). Other studies related the local production of RA-specific antibodies to an observed higher level of specific antibodies in SF and synovial tissue over plasma in the same patient (50, 51).

To evaluate conceivable differences between individual antibody clones or the lower abundant RA-specific ACPA subrepertoire, we therefore resolved the molecular diversity of paired total and ACPA IgG1 repertoires and investigated the abundance of the detected highest abundant Fab molecules using total and antigen-specific Fab profiling (22, 23). Strikingly, neither a substantial enrichment of high abundant total IgG1 Fab molecules was determined nor were differences in the detected dominant ACPA IgG1 Fab molecules observed. In contrast, total plasma and SF IgG1 repertoires showed a very good correlation, and even the ACPA IgG1 Fab repertoires were highly alike in the paired samples. Although the precise location of antibody production cannot be determined with certainty and fluid-specific modifications of antibody clones, for example, because of local differences in glycosidase and protease activities may occur (49, 55, 56), our data thus reveal that not only the total plasma proteome but also the total IgG1 as well as the markedly lower abundant IgG1 (auto)antibody clones found in plasma are similarly reflected in the SFs of RA patients.

While early reports had suggested major differences in the filtration of plasma proteins from blood into SF of “healthy” joints depending on the molecular dimensions of the transported proteins, our here presented work thus provides a comprehensive dataset, revealing that—in swollen joints of RA patients—no major filtering seems to occur. Instead, apparently all plasma proteins pass into SF without substantial bias induced by the synovial lining. With regard to systemically administered therapeutics, it is thus likely that no major differences in biodistribution into swollen joints of RA patients occur, and at least protein-based therapeutics are equally delivered independent of their abundance, size, charge, or other biochemical and biophysical properties. Notably, this finding is also highly relevant for pharmacokinetic studies of such therapeutics, as determining the quantity of therapeutics directly in SF comes with major challenges. Our findings indicate that the concentrations of proteins, and hence likely also protein-based therapeutics, in blood directly reflect to those entering the SF of the studied joints in RA patients. We therefore propose that pharmacokinetic analyses of (protein-based) therapeutics in blood are applicable to assess their biodistribution into swollen joints of RA patients. The difference in permeability of “healthy” and swollen joints for large proteins may furthermore serve as tool to preferentially deliver therapeutics into swollen joints while sparing “healthy” ones or to restrict the penetration of a therapeutic once joint inflammation has been resolved.

An altered permeability of the synovial tissue for plasma proteins may, however, also have direct consequences for joint functioning. Next to the degradation of factors responsible for the physiological functions of SF, such as lubricin or hyaluronan, by enzymes or radicals released upon local inflammation (57, 58, 59), also an enhanced influx of plasma consequent to the increased permeability contributes to the reduced concentrations of these factors observed for SF of RA patients (60), eventually diminishing the physiological functions of SF and likely resulting in impaired joint functioning. Together with the local (immune) processes mirrored in the SF proteomes, our multilayer data thus not only served to revisit the filtration from blood to SF suggested already several decades ago; it also exemplifies the use of in-depth proteomic analyses of SF to monitor and evaluate the local (immune) processes connected to RA pathology.

## Data availability

The raw LC–MS/MS files and peptide–protein identification have been deposited to the ProteomeXchange Consortium *via* the PRIDE partner repository with the dataset identifier PXD054336.

## Supplemental data

This article contains supplemental data (61, 62, 63, 64, 65, 66).

## Conflict of interest

The authors declare no competing interests.

## Supplemental Data

Supplemental Table S1A

Supplemental Table S1B
