# Supplementary material for: Multitiered Proteome Analysis Displays the Hyperpermeability of the Rheumatoid Synovial Compartment for Plasma Proteins
Source: Mol Cell Proteomics. 2024 Dec 31;24(2):100900. doi: 10.1016/j.mcpro.2024.100900 (PMC11821404; doi:10.1016/j.mcpro.2024.100900)
Supplement: Supplemental Data [file mmc4.pdf]

Supporting Information

Multitiered proteome analysis displays the hyperpermeability of the rheumatoid synovial compartment for plasma proteins

Eva Maria Stork[1,#], Sofia Kalaidopoulou Nteak[2,3,#], Danique M.H. van Rijswijck[2,3,#], J. Mirjam A. Damen[2,3], Hans Ulrich Scherer[1], Rene E.M. Toes[1], Albert Bondt[2,3], Tom W.J. Huizinga[1] and Albert J.R. Heck[2,3,*]

[1] Department of Rheumatology, Leiden University Medical Center, Albinusdreef 2, 2333 ZA Leiden, The Netherlands
[2] Biomolecular Mass Spectrometry and Proteomics, Bijvoet Center for Biomolecular Research and Utrecht Institute for Pharmaceutical Sciences, University of Utrecht, Padualaan 8, Utrecht 3584 CH, The Netherlands
[3] Netherlands Proteomics Center, Padualaan 8, Utrecht 3584 CH, the Netherlands
[#] These authors contributed equally to this work and share first authorship

[*] Correspondence to Albert J.R. Heck (a.j.r.heck@uu.nl)

Table of Contents

	Page
**Supplemental Figure S1 | Quantitative overview of the relative abundance of the ~560 most abundant proteins.**	S3
**Supplemental Figure S2 | Protein abundance of specific proteins highly enriched in SF.**	S4
**Supplemental Figure S3 | High correlation of 19 selected plasma proteins of diverse molecular weight.**	S5
**Supplemental Figure S4 | Total plasma and SF IgG1 Fab profiles of each individual RA patient.**	S6
**Supplemental Figure S5 | Plasma and SF ACPA IgG1 Fab profiles obtained for plasma and synovial fluid of patients 01, 02, 04 and 08.**	S7
**Supplemental Table 1a | Total plasma and SF protein abundances across all samples.**	xlsx
**Supplemental Table 1b | Selected plasma and SF protein abundances across all samples.**	xlsx
**Supplemental Table 2 | 19 selected plasma proteins with their molecular weights.**	xlsx

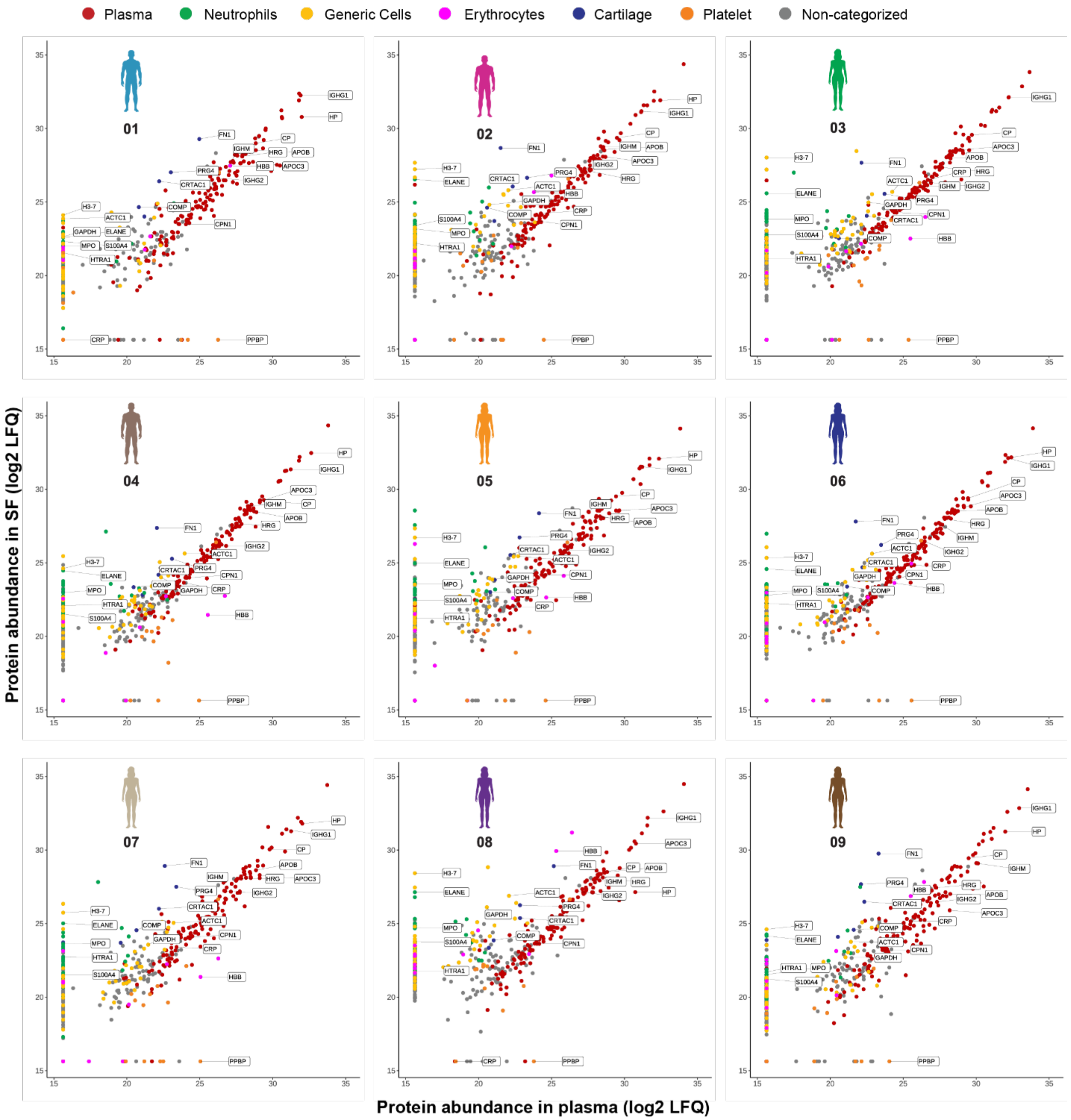

Supplemental Figure S1. Quantitative overview of the relative abundance of the ~560 most abundant proteins. Comparing their abundance (as estimated based on LFQ values) in plasma versus SF of each of the n=9 RA patients studied. Genuine plasma proteins are annotated in red. Proteins related to cartilage/chondrocytes, erythrocytes, neutrophils, platelets and generic, i.e. unspecified, cells are annotated in blue, pink, green, orange and yellow, respectively. Proteins that could not be undoubtedly classified are indicated in gray. If a protein was not detected in one of the body fluids, a minimal LFQ value of 16 was imputed.

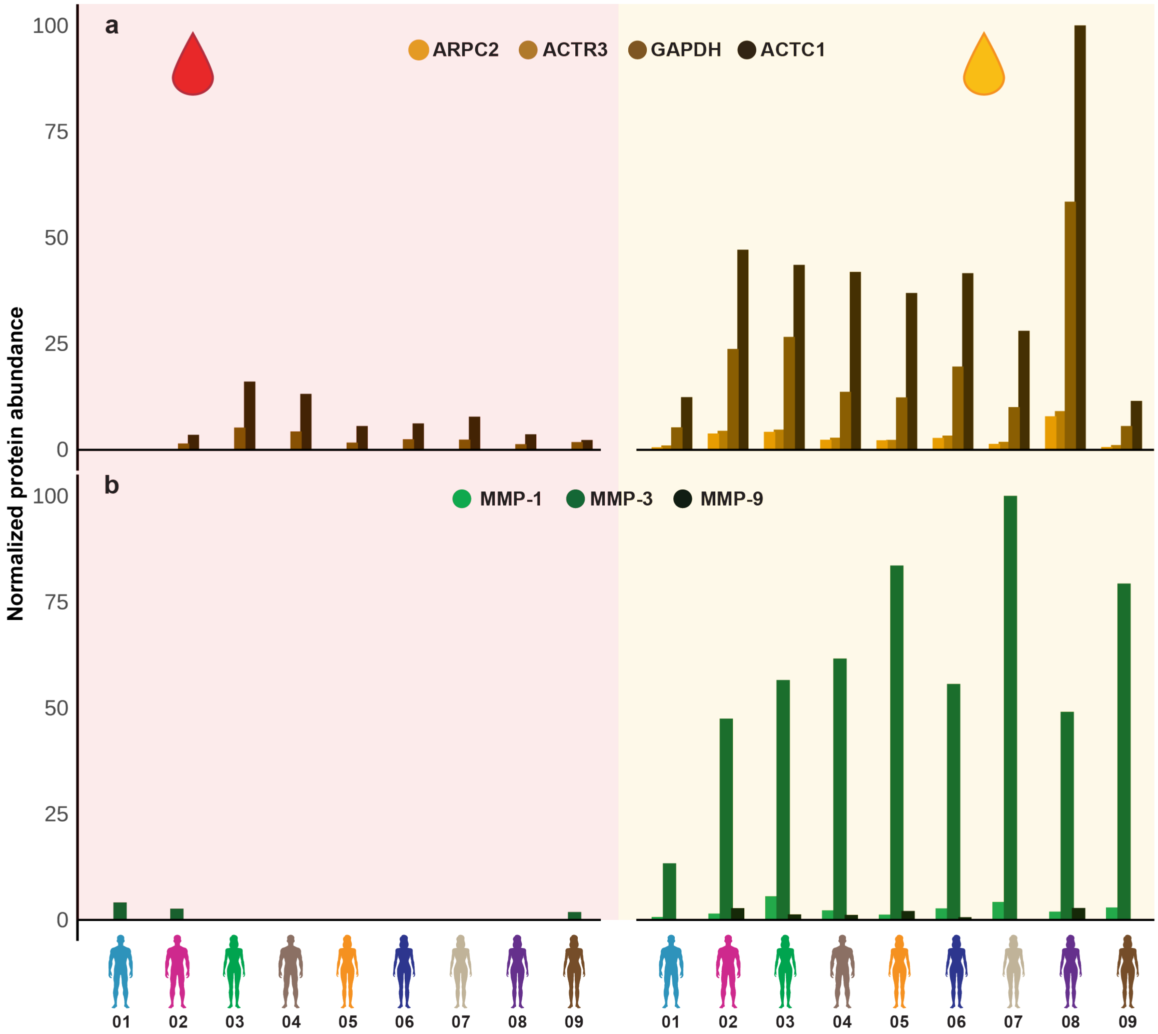

Supplemental Figure S2. Normalized protein abundance of specific proteins highly enriched in SF. The actins (ARPC2, ACTR3, ACTC1) and GAPDH in **(a)** are detected mainly in SF whereas their abundance in plasma is low to non-detectable. The same is observed for MMPs **(b)**, especially MMP-3 which is very abundant in SF whereas in plasma it is only found at very low concentrations in patients 01, 02 and 09. The LFQ values were normalized by subtracting the minimal from the original LFQ value and dividing by the difference of maximal and minimal LFQ.

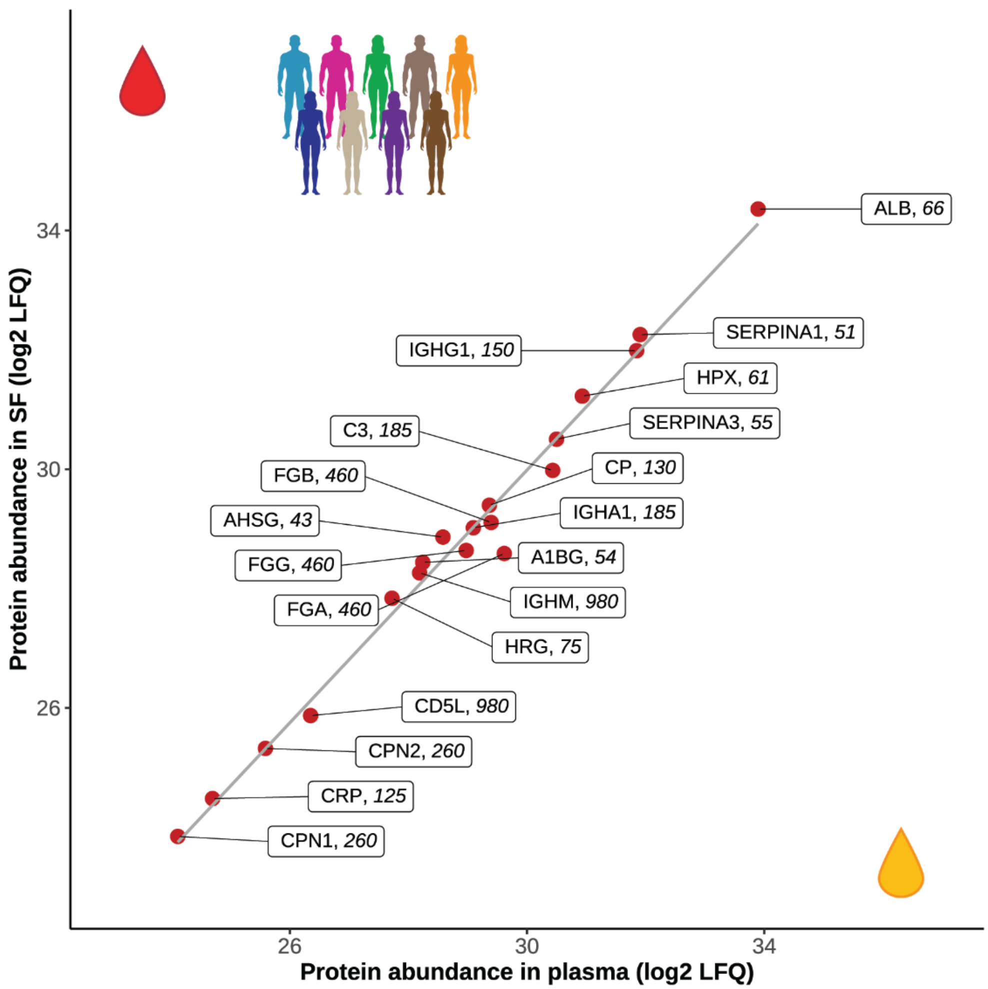

Supplemental Figure S3. Correlation of 19 selected plasma proteins of diverse molecular weight. Each dot represents one protein and is labelled by the protein name followed by the molecular weight in kDa (italics) of the monomer or polymer, accordingly. The depicted proteins were selected to cover a range of molecular weight from low (~40 to 100 kDa) over medium (~110 to 500 kDa) to high (~510 to 1000 kDa) molecular weight. Of note, the abundance of the depicted proteins in plasma and SF highly correlates independent of the size of the respective protein as indicated by the grey fitted line.

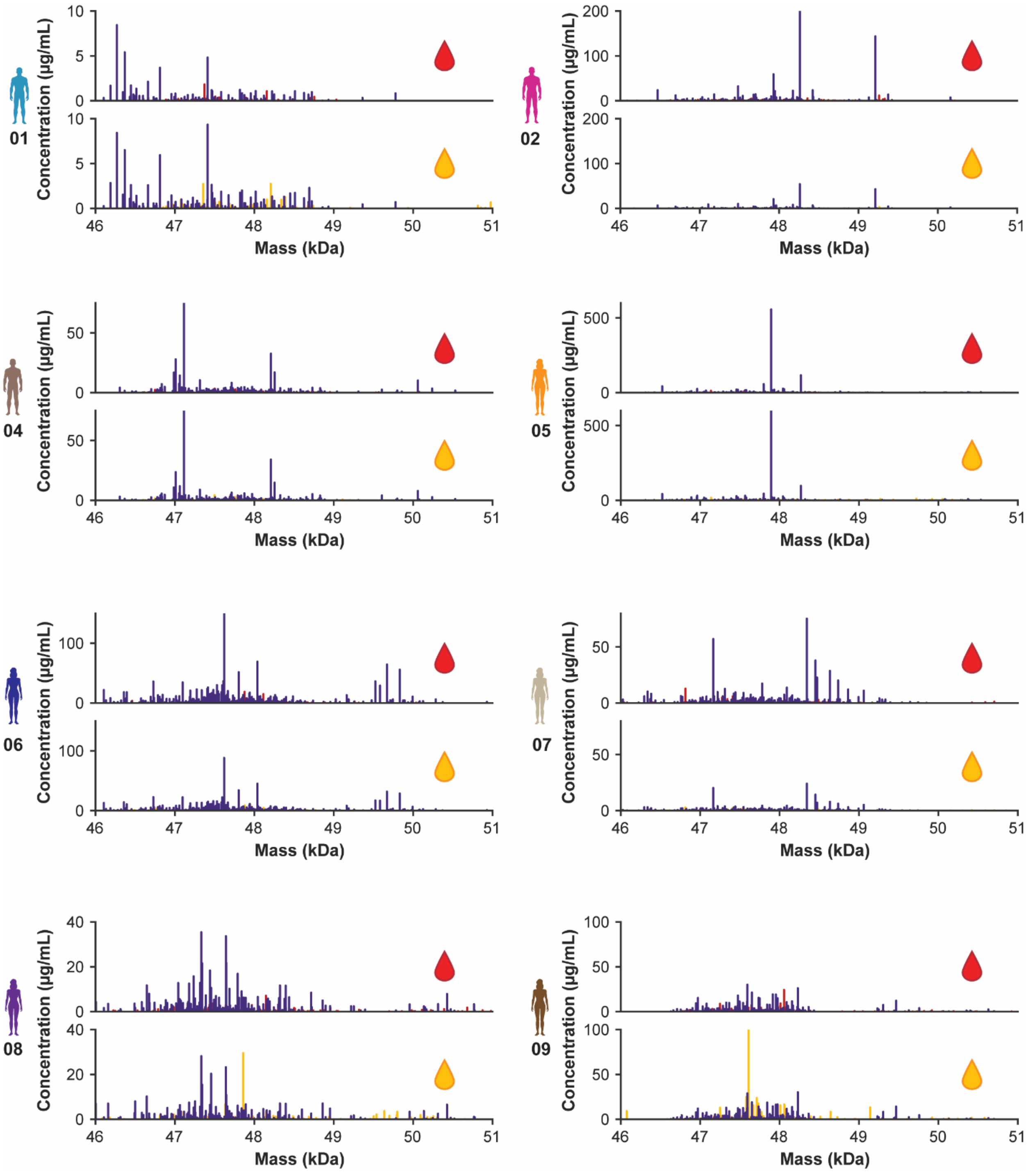

Supplemental Figure S4. Total plasma and SF IgG1 Fab profiles of each individual RA patient. For each patient, the total plasma (top row) and total SF (bottom row) are shown. Y-axes are scaled based on the concentration of the highest abundant Fab molecule of the individual's IgG1 Fab profile. Fab molecules shared between both fluids are depicted in purple, Fab molecules uniquely detected in plasma or SF are depicted in red and yellow, respectively. Of note, the total plasma IgG1 Fab profile of patient 03 is shown as an example in Figure 5.

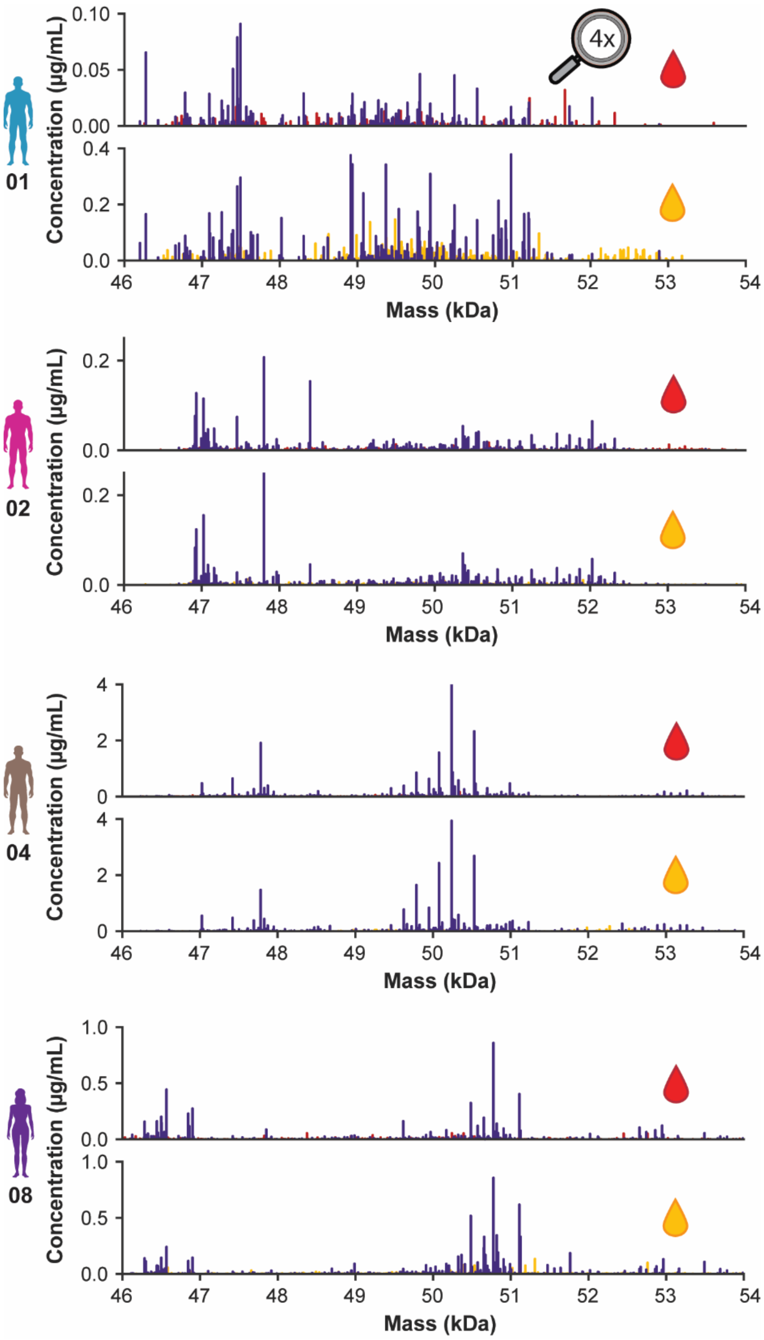

Supplemental Figure S5. Plasma and SF ACPA IgG1 Fab profiles obtained for patients 01, 02, 04, and 08. Fab molecules shared between both fluids are depicted in purple, Fab molecules uniquely detected in plasma or synovial fluid are depicted in red and yellow, respectively. Of note, ACPA IgG1 Fab profiles of patients 02 and 04 are also shown as examples in Figure 5. Notably, due to ACPA Fab glycosylation the mass of many ACPA Fab molecules is distinctively higher (i.e. >49 kDa).
